# Reliability of a two-probe ultrasound imaging procedure to measure strain in the Achilles tendon

**DOI:** 10.1186/s13047-019-0358-6

**Published:** 2019-09-18

**Authors:** Prue Molyneux, Richard F. Ellis, Matthew Carroll

**Affiliations:** 1 Department of Podiatry, School of Clinical Sciences, Faculty of Health & Environmental Sciences, Auckland University of Technology, Private Bag 92006, Auckland, 1142 New Zealand; 2 Department of Physiotherapy, School of Clinical Sciences, Faculty of Health & Environmental Sciences, Auckland University of Technology, Private Bag 92006, Auckland, 1142 New Zealand

**Keywords:** Achilles tendon, Ultrasound imaging, Reliability

## Abstract

**Background:**

Alteration in the strain properties of the Achilles tendon may lead to adaptations such as pathological stiffening. Stiff tendons have reduced adaptive ability, which may increase the risk for developing tendinopathy. Strain can be measured using musculoskeletal ultrasound imaging. A two-probe ultrasound procedure may reduce the measurement error associated with a one-probe procedure. However, the reliability of the two-probe procedure has not been established. This study aimed to determine the within-session intra- and inter-rater reliability and between-session reliability of a two-probe ultrasound procedure to measure Achilles tendon strain.

**Methods:**

Participants were 29 healthy individuals (19 females, 10 males; mean age 33.6 years). Achilles tendon images were acquired with a two-probe ultrasound procedure as the ankle moved through a standardised range of motion (20° plantarflexion to 10° dorsiflexion). Both probes were positioned longitudinally, one over the musculotendinous junction and the second over the calcaneal insertion of the Achilles tendon. Repeat measurements were taken for all participants at the initial study visit, and for 10 participants in a second measurement session 4 weeks later. Strain measures were calculated from pre-captured images using Motion Analysis 2014v1 software by two independent raters. Within-session intra- and inter-rater reliability and between-session intra-rater reliability were calculated using intraclass correlation coefficients (ICC) with 95% confidence intervals. The standard error of measurement was also calculated.

**Results:**

The two-probe procedure to measure Achilles tendon strain showed excellent within-session intra-rater (ICC = 0.84, *p* < 0.001) and inter-rater reliability (ICC = 0.88, *p* = 0.003), but poor between-session intra-rater reliability (ICC = 0.18, *p* = 0.397).

**Conclusion:**

The two-probe procedure to measure Achilles tendon strain is reliable for repeated measurements on the same day. However, measurement error increased when strain was measured on different days, which may be attributable to a combination of examiner error and participant factors. Measurement of Achilles tendon strain offers an additional tool for evaluating the tendon’s mechanical characteristics. The ability to reliably quantify strain may allow clinicians to identify those at risk for Achilles tendinopathy and formulate more effective management plans.

## Background

Strain is a measure of mechanical deformation and is defined as the percent of elongation of a structure [1]. Strain is measured by the displacement or elongation of the structure of interest (i.e. Achilles tendon) relative to its resting length [2, 3]. Achilles tendon injuries are the most prevalent overuse injury of the lower limb [4, 5]. Kujala et al. [6] reported that one in two professional runners will experience Achilles tendinopathy before age 45 years compared with one in 10 people in the general population. Achilles tendinopathy is characterised by pain and swelling in and around the Achilles tendon, accompanied by impaired physical function [7]. Alteration in the strain properties of the Achilles tendon induced by mechanical loading is associated with risk for injury and long-term tendon adaptation, such as pathological stiffening [3, 8, 9]. Stiff tendons have less adaptive ability, which may predispose to the development of tendinopathy at relatively lower tendon loads [10].

A common method used for in-vivo strain calculation in the Achilles tendon is visualisation of tendon excursion using a single-probe ultrasound procedure [3, 11]. A single-probe procedure requires the use of a marker on the skin to cast an acoustic shadow into the field of view [12, 13]. This shadow is used as a reference point to measure change in the length of the tendon, enabling calculation of strain. A limitation of the single-probe procedure is the possibility of surface marker movement during the testing procedure as a result of skin displacement [14]. This may influence the calculation of tendon strain by producing an inaccurate measure of tendon resting length [15, 16]. A two-probe procedure eliminates problems caused by the use of skin markers and probe relocation. Furthermore, the two probe positions are fixed, which allows strain measurement to be calculated from assessment of the difference in tendon movement over a known distance.

Two previous studies have investigated the measurement of Achilles tendon strain using a two-probe procedure [17, 18]. Arampatzis et al. [17] conducted an in-vivo examination of the elongation and strain of the gastrocnemius medialis tendon and aponeurosis during maximal voluntary plantarflexion effort in 12 sprinters. They reported the maximal strain of the gastrocnemius medialis tendon and aponeurosis were 4.7 and 5.1%, respectively. Neugebauer and Dawkins (18) assessed differences in Achilles tendon strain during an isometric plantarflexion exercise after 6 months of growth among children aged 10–14 years. They found that mean Achilles tendon strain (3.8%) did not differ between testing sessions or by sex. Although both studies quantified strain, no previous research has assessed the within or between session reliability of the two-probe procedure.

This study aimed to quantify Achilles tendon strain during passive tendon elongation using a two-probe ultrasound procedure, and investigate the intra- and inter-rater within-session reliability and inter-rater between-session reliability of the two-probe procedure.

## Methods

### Participants

Participants were 29 healthy volunteers (19 females, 10 males). Most (*n* = 23) were European, two were Māori, and four were Asian. Participants’ mean (standard deviation [SD]) age was 33.6 (12.5) years, and the mean (SD) body mass index was 26.4 (5.3) kg/m^2^. The inclusion criteria were individuals aged 18–65 years with no self-reported history of Achilles tendon injury. Participants were excluded if they had: a history of Achilles tendon pain; previous injury to the gastrocnemius; concomitant injury or pain developing from structures other than the Achilles tendon in the lower limb (e.g. plantar heel pain); inflammatory arthritis or a neurological, metabolic or endocrine disorder; a history of Achilles tendon rupture; or previous lower limb trauma, surgery or corticosteroid injection at the Achilles tendon. This study was approved by the Auckland University of Technology Ethics Committee (AUTEC 17/429). All participants provided written informed consent before entry into the study.

### Experimental protocol

**Fig. 1:**
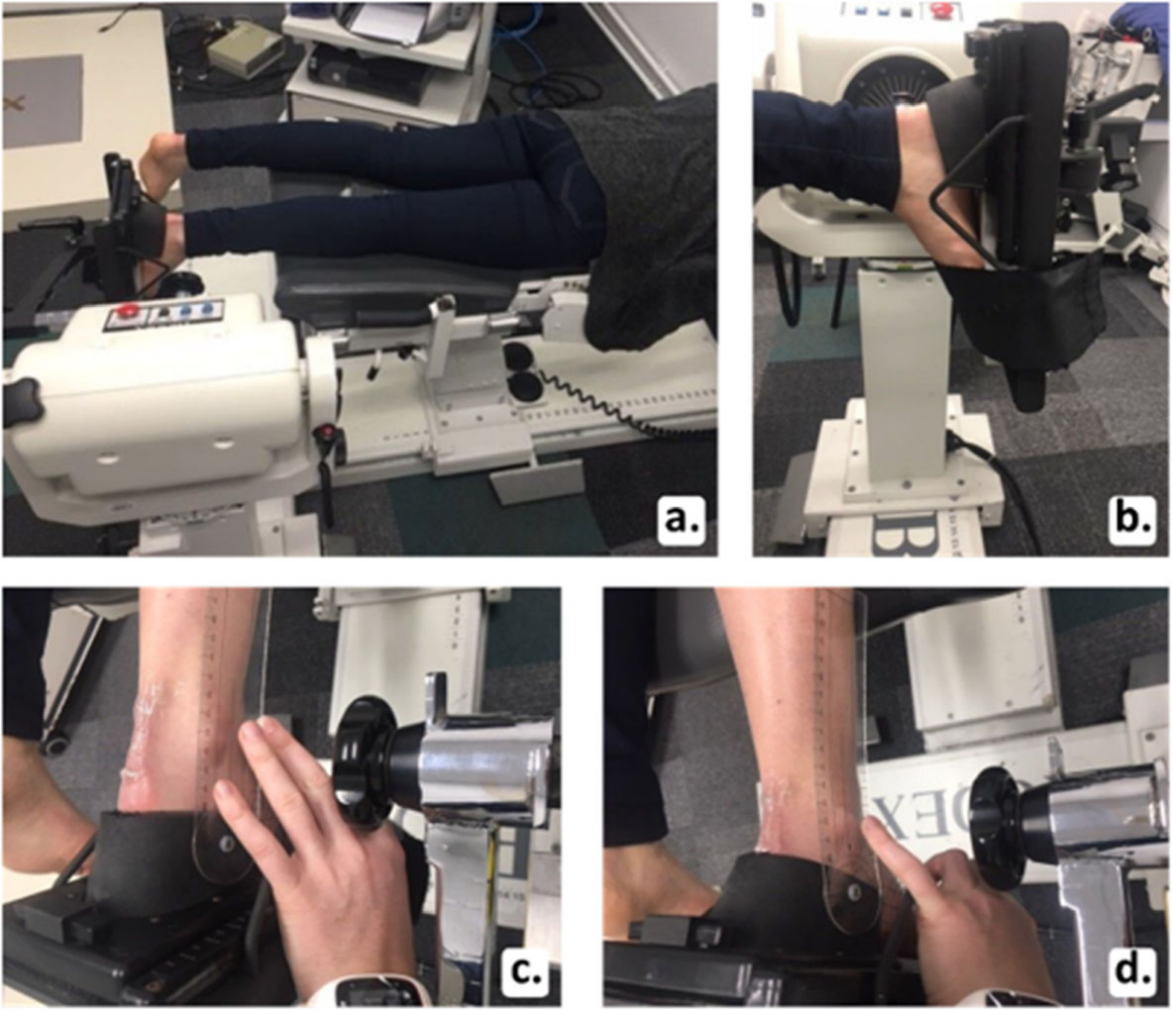
Participant positioning on Biodex machine (**a**); foot positioning on foot plate (**b**); 10° of ankle joint dorsiflexion (**c**); 20° of ankle joint plantarflexion (**d**)

Two identical portable Terason 3300 ultrasound machines (Terason, Teratech Corporation, Burlington, MA) were used to acquire all image sequences. Both machines were equipped with a 12–5 MHz 50 mm linear-array transducer. Foot position and ankle motion were standardised with the use of the Biodex system 4 PRO (Biodex Medical Systems, Inc., Shirley, New York). Participants lay in a prone position on the Biodex with the right knee extended and the ankle fixed at 90° flexion, with the lateral malleolus aligned with the Biodex axis of rotation (Fig. 1a and b). Joint range of motion was set using a manual goniometer; the ankle was first positioned in 10° of ankle joint dorsiflexion (Fig. 1c), and then 20° of ankle joint plantarflexion (Fig. 1d). Away and toward range of motion limits were set enabling the Biodex machine to move the ankle from 20° plantarflexion to 10° dorsiflexion in one movement sequence.

### Ultrasound image acquisition

**Fig. 2:**
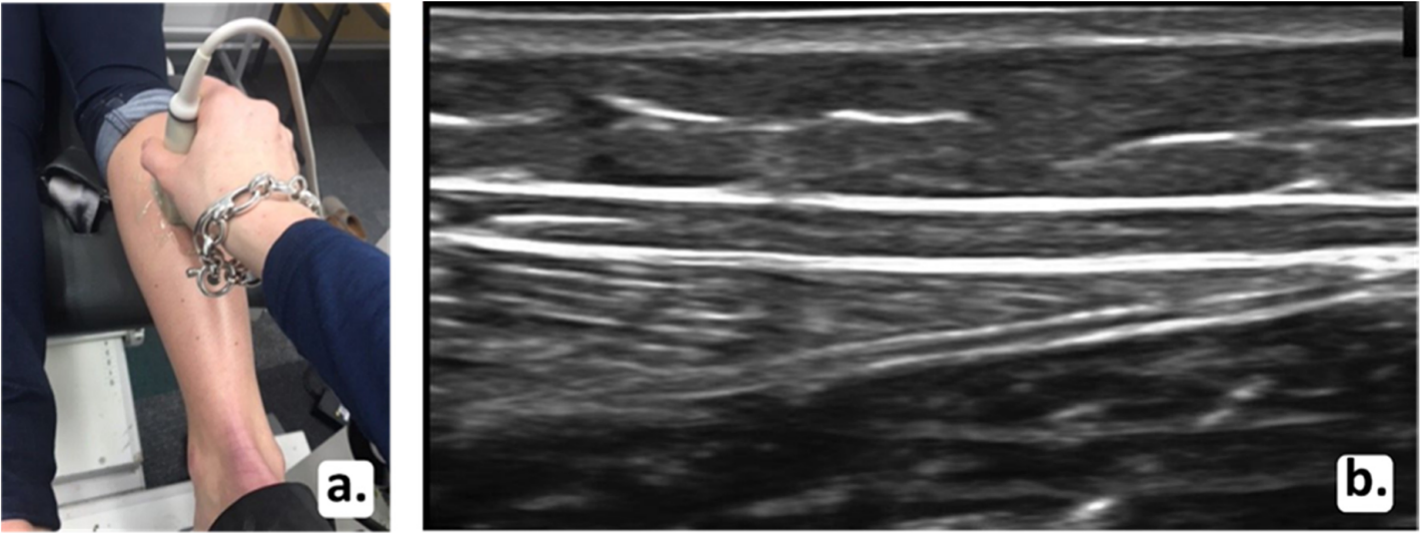
First probe positioning (**a**) and resulting ultrasound image of musculotendinous junction as indicated by the arrow (**b**)

**Fig. 3:**
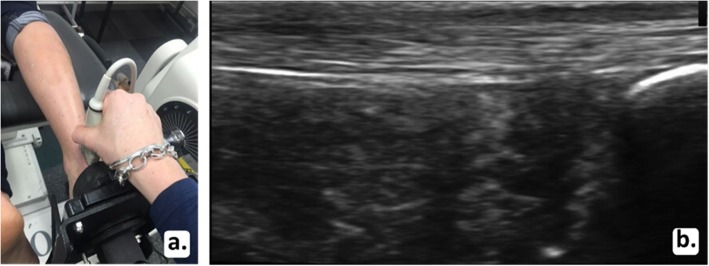
Second probe positioning (**a**) and resulting ultrasound image of calcaneal insertion of the Achilles tendon as indicated by the arrow (**b**)

**Fig. 4:**
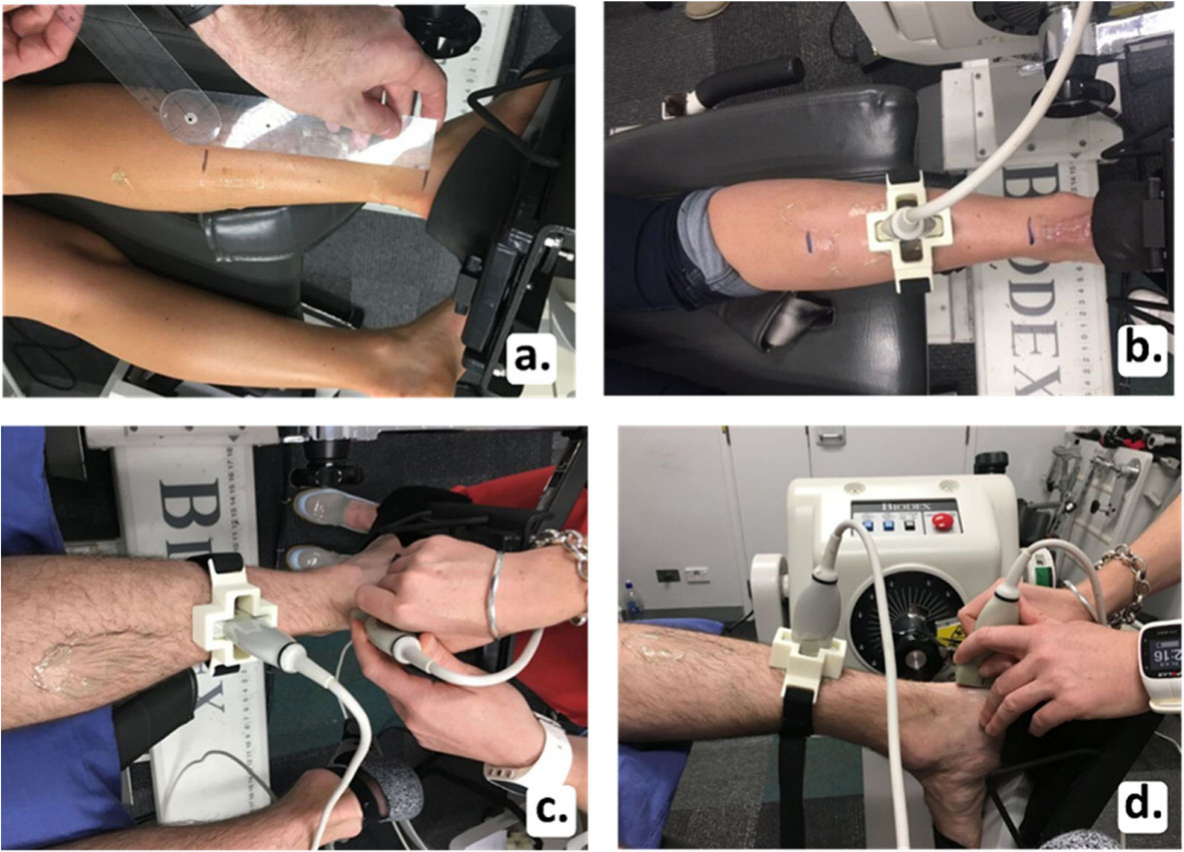
Measurement of resting length (**a**); first probe positioning at musculotendinous junction (**b**) and second probe being held by the researcher capturing the calcaneal insertion (**c** and **d**)

The two Terason ultrasound machines were set up identically. The right leg was imaged in all participants using B-mode imaging with both ultrasound probes positioned in the longitudinal plane by one examiner (PM). The machine was set with one focal zone; the depth was 2 cm and the gain was adjusted to ensure a clear image was obtained. The depth was adjusted to 3 cm for two participants who had greater subcutaneous fat in their calf region, which required deeper penetration of the ultrasound to capture the Achilles tendon. One ultrasound probe was used to identify the medial head of the gastrocnemius (Fig. 2a). The probe was then moved distally until the musculotendinous junction (MTJ) was just visible on the right side of the ultrasound screen (Fig. 2b). The second probe was used to capture the central region of the Achilles tendon insertion (Fig. 3a). This ultrasound probe was used to identify the calcaneal insertion of the Achilles tendon and was moved proximally so the most distal point of the tendon on the calcaneus was visible on the right side of the second ultrasound screen (Fig. 3b). These probe placement locations were chosen for two reasons. First, it was necessary to have two clearly defined and consistent anatomical locations to ensure standardisation of measurements. Visualisation of and placement at the MTJ and tendon insertion allowed this standardisation. Second, the two locations allowed full consideration of the tendon along its length from the insertion to the MTJ. To measure tendon elongation, the skin was marked where the proximal end of each ultrasound probe reached. The distance between these two points was recorded as the resting length in millimetres (Fig. 4a). To capture tendon elongation at the MTJ, the first probe was held in a customised probe holder and secured with a Velcro strap positioned halfway between these markers (Fig. 4b). The MTJ probe was positioned between these two marks so the MTJ remained in the field of view on the ultrasound screen during tendon excursion. The second probe capturing the Achilles tendon insertion was held by the researcher (Fig. 4c and d).

### Image acquisition

Each participant had a standardised warm-up of three repetitions of ankle joint movement from plantarflexion to dorsiflexion. Once the ultrasound probes were positioned, the Biodex moved the ankle through the passive end range limits from 20° plantarflexion to 10° dorsiflexion at five degrees per second to capture Achilles tendon elongation. A video sequence of tendon excursion in the longitudinal plane was recorded and four quality sequences from each probe were obtained. The two best video sequences were selected for analysis and named Trial 1 and Trial 2. The selected sequences had to be the matched sequence for both MTJ and insertion. The video sequence was captured over a 5-s period at a capture rate of 30 frames per second. All videos were acquired by a podiatrist (PM), who had attended specialised imaging training workshops and received supervised education regarding ultrasound imaging and image motion analysis software by an experienced musculoskeletal ultrasonographer (RE).

### Image motion analysis and calculation software

**Fig. 5:**
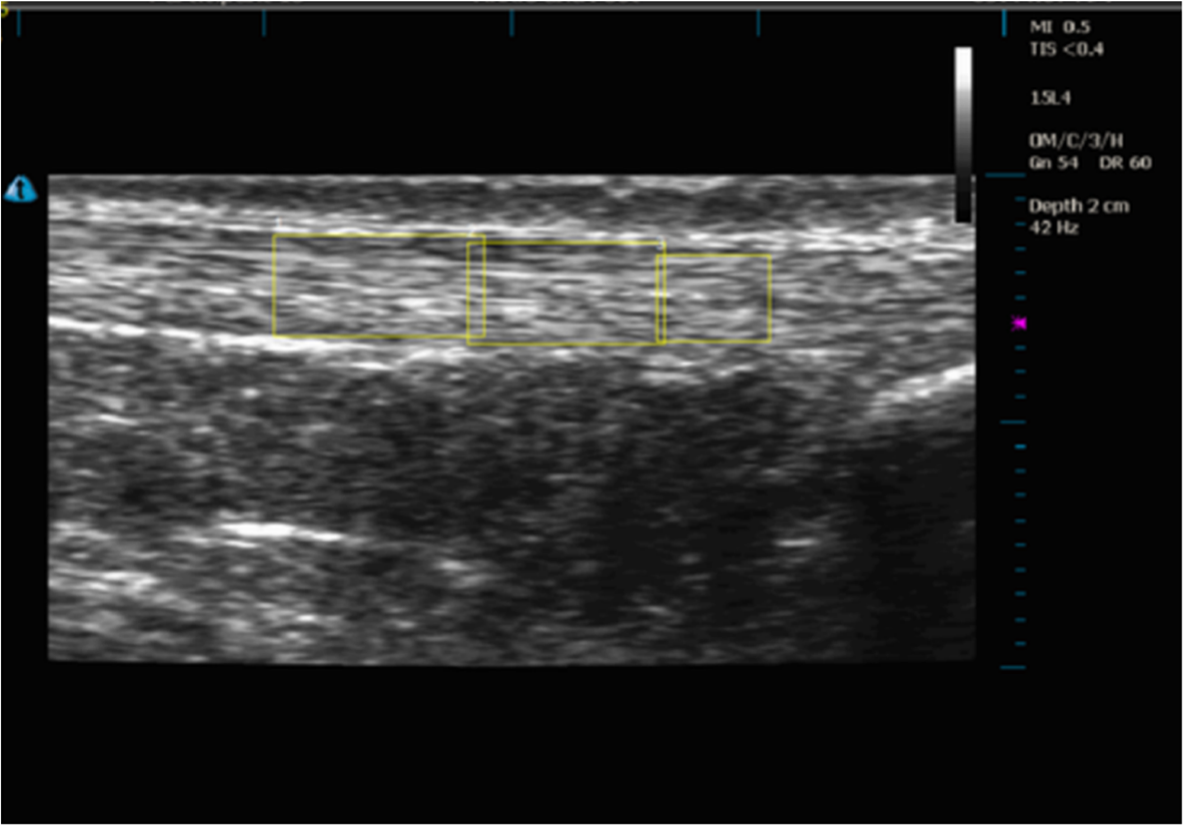
Regions of interest within the Achilles tendon

First, each image sequence was analysed to obtain Achilles tendon displacement measures. The researcher used frame-by-frame cross correlation analysis to measure longitudinal movement of the Achilles tendon using software developed in MAT-lab (Mathworks, Natick, MA, USA) by Dilley et al. [19]. This software has been successfully used to measure tendon excursion [20]. This method uses a cross-correlation algorithm to determine relative tissue movement (in this case, tendon) between successive frames in a sequence of ultrasound images [19]. ImageJ (National Institutes of Health, Bethesda, MA, USA) was then used to calculate the resolution of the ultrasound image and determine a scale conversion for pixels to millimetres. To analyse the movement of the tendon, three contiguous rectangular regions of interest (ROI) of varied dimensions within the Achilles tendon were selected within a predetermined range (Fig. 5). The predetermined range was standardised through division lines on the ultrasound screen that enabled the image to be split into quarters. The ROI was standardised in the middle half of the divisions for all images at the MTJ and insertion.

The software compares gray-scale values of speckle features from the ROI between adjacent frames of the image sequence by a correlation coefficient calculation for each individual pixel shift [19]. MAT-lab also takes into account any potential movement of the skin relative to the ultrasound probe. Pixel shift measurements within the background ultrasound field from stationary structures (e.g. subcutaneous tissue) were subtracted from the pixel shift measurements from the tendon. This method provides a specific calculation of tendon elongation by removing potential error that may arise from any relative movement of the ultrasound probe.

### Data analysis

Raw data were described as mean (SD) for continuous variables or n (%) for categorical variables. All data analysis was conducted using SPSS version 24 (SPSS Inc., Chicago, IL, USA) with an alpha level of *p* < 0.05. Strain (%) was calculated using the following equation, as described by Dilley et al. [19].

$$ \mathrm{Strain}\%=\frac{\mathrm{MTJ}\ \mathrm{excursion}\ \left(\mathrm{mm}\right)-\mathrm{insertion}\ \mathrm{excursion}\ \left(\mathrm{mm}\right)\ \mathrm{x}\ 100}{\mathrm{Achilles}\ \mathrm{tendon}\ \mathrm{resting}\ \mathrm{length}} $$

Ultrasound images for Trials 1 and 2 were obtained in a single session by a single assessor (PM) to allow assessment of within-session intra-rater reliability. This was calculated using a two-way random, single measures intraclass correlation coefficient (ICC) (3, 1), with absolute agreement [21]. In addition, we calculated 95% confidence intervals (CI) for the ICC [21], standard error of measurement (SEM) [22] and minimal detectable change (MDC) [23].

To determine between-session intra-rater reliability, 10 participants were randomly-selected by a random number generator to attend a second testing session 4 weeks after the initial measurement session. Reliability was calculated using a two-way mixed average measures ICC (3, 1) with absolute agreement [24], and 95% CIs, SEM and MDC values were calculated.

For the total sample (29 participants), 10 pre-captured images were randomly selected to determine the within-session inter-rater reliability of the motion analysis software. Tendon excursions were compared between the first rater (PM) and a second rater (RE), who had expertise in the use of the motion analysis software and was blinded to the results. A two-way mixed average measures ICC (3, 2) with consistency [21] was calculated along with 95% CIs, SEM and MDC values to determine inter-rater reliability.

## Results

### Intra-rater within-session reliability

Descriptive statistics for Achilles tendon excursion at the MTJ and insertion and the distance between two probes (resting length) for Trials 1 and 2 are presented in Table 1. The ICC revealed excellent within-session intra-rater reliability for the assessment of Achilles tendon strain (ICC = 0.84), with mean strain values of 4.93 and 5.34% for Trials 1 and 2, respectively (Table 2). The excellent levels of reliability were confirmed by the small measurement error, with SEM values of 1.07 and 1.26% for Trials 1 and 2, respectively. MDC values were 2.86% for Trial 1 and 3.11% for Trial 2.

**Table 1:** Achilles tendon excursion measurements at the musculotendinous junction and calcaneal insertion between Trial 1 and Trial 2

	MTJ, mm mean (SD)	Insertion, mm mean (SD)	Distance, mm mean (SD)
Trial 1	5.04 (2.7)	0.17 (1.6)	100.8 (12.9)
Trial 2	5.21 (2.6)	−0.23 (1.7)	100.8 (12.9)

*MTJ* musculotendinous junction, *SD* standard deviation

**Table 2:** Strain and intra-class correlation coefficients between Trial 1 and Trial 2

	Strain (%) mean (SD)	ICC	*P*	95% CI	SEM	MDC
Trial 1	4.93 (2.67)	0.84	< 0.001	0.69–0.92	1.07	2.86
Trial 2	5.34 (3.14)				1.26	3.11

*SD* standard deviation, *ICC* intraclass correlation coefficient, *CI* confidence interval, *SEM* standard error of measurement, *MCD* minimal detectable change

### Intra-rater between-session reliability

Ten participants attended a second study visit to assess the inter-session reliability of the two-probe method of assessing Achilles tendon strain. Descriptive statistics for the Achilles tendon excursion at the MTJ and insertion and the distance between two probes (resting length) for Sessions 1 and 2 are presented in Table 3. The ICC revealed poor between-session intra-rater reliability (ICC = 0.18) (Table 4). The SEM values were 2.38 and 3.99% for Sessions 1 and 2, respectively. The MDC values were 4.28 and 5.54% for Sessions 1 and 2, respectively. There was no statistically significant difference in the mean strain values between Sessions 1 and 2 (*P* = 0.397).

**Table 3:** Achilles tendon excursion measurements at the musculotendinous junction and calcaneal insertion between Session 1 and Session 2 (*n* = 10)

	MTJ, mm mean (SD)	Insertion, mm mean (SD)	Distance, mm mean (SD)
Session 1	7.12 (3.61)	0.32 (1.49)	105.45 (14.16)
Session 2	6.41 (2.91)	−0.48 (1.99)	102.20 (14.94)

*MTJ* musculotendinous junction, *mm* millimetres, *SD* standard deviation

**Table 4:** Strain and intra-class correlation coefficients between Session 1 and Session 2

	Strain (%) mean (SD)	ICC	*P*	95% CI	SEM	MCD
Session 1	6.60 (2.63)	0.18	0.397	−3.49–0.81	2.38	4.28
Session 2	6.69 (4.41)				3.99	5.54

*SD* standard deviation, *ICC* intraclass correlation coefficient, *CI* confidence intervals, *SEM* standard error of measurement, *MCD* minimal detectable change

### Inter-rater within-session reliability

The data revealed a statistically significant difference between the mean strain values for Raters 1 and 2 (*P* = 0.003). However, the inter-rater within-session reliability was excellent (ICC = 0.88) with low measurement error (Table 5).

**Table 5:** Strain and intra-class correlation coefficients between Rater 1 and Rater 2

	Strain (%) mean (SD)	ICC	*P*	95% CI	SEM	MCD
Rater 1	5.89 (3.52)	0.88	0.003	0.48–0.97	1.22	3.06
Rater 2	5.67 (2.96)				1.03	2.81

*SD* standard deviation, *ICC* intraclass correlation coefficient, *CI* confidence intervals, *SEM* standard error of measurement, *MCD* minimal detectable change

## Discussion

Data from the present study indicated that the two-probe ultrasound procedure to measure strain in the Achilles tendon had excellent within-session intra-rater and inter-rater reliability, but poor between-session intra-rater reliability. The excellent within-session reliability with small measurement error may be attributable to the standardised methodology used in the within-session trials. This included standardisation of participants’ positioning during image acquisition, use of the Biodex machine to ensure coordinated and consistent ankle movement and use of a custom-built ultrasound probe holder, removing the need for manual probe repositioning. The excellent within-session inter-rater reliability indicated that both raters were consistent with the use of the MAT-lab and ImageJ software. The within-session inter-rater reliability only referred to the image motion analysis and calculation software part of the present study’s methodology. These results were consistent with other studies using this software, which demonstrated high reliability in the assessment of nerve excursion [19, 25, 26].

The poor between-session intra-rater reliability may be attributable to participant and probe positioning factors. Although participants’ positioning and the experimental setup were standardised, the between-session experimental positioning might have varied in a number of ways, including: 1) variation in defining ankle range of motion limits, 2) variation in probe positioning as the skin was re-marked and probes repositioned between sessions and 3) variation due to non-standardisation of activity levels before measurement. Activity levels before strain measurement have been shown to alter strain values in the Achilles tendon [14, 27]. Activity levels may affect the viscoelasticity of the Achilles tendon, potentially influencing the strain measurement.

Comparison of data from the present study with previous research using a two-probe procedure is problematic because of numerous methodological variations between the studies, including: a) age-related differences, b) probe placement variation, c) differing activation procedures and d) differences in determining ankle joint range of motion parameters.

### Age-related differences

The mean strain values in the present study (5.14% Session 1 and 6.65% Session 2) were higher than those reported by Arampatzis et al. [17] (mean strain 4.70%) and Neugebauer and Dawkins [18] (mean strain 3.80%). Arampatzis et al. [17] assessed Achilles tendon strain in participants with a mean age of 19.6 years, and Neugebauer and Dawkins [18] included participants with a mean age of 12.3 years. Although previous data suggested strain was reduced with increasing age [16, 28, 29], data from the present study (participants’ mean age 33.6 years) contradicted these findings.

### Variation in probe placement

There was clear variation in the location of ultrasound probes between the present study and previous studies. In this study, one probe was fixed and that the other was repositioned at the MTJ. In contrast, the probe placement in Arampatzis et al. [17] was not directly on the Achilles tendon; one probe was positioned to visualise the Achilles tendon at the MTJ and the second was positioned to visualise the aponeurosis of the gastrocnemius medialis [17]. The rationale for this positioning was based on conflicting work that showed strain of the tendon and the aponeurosis was heterogeneously distributed along their length and that strain was greater for the aponeurosis [30–33]. Arampatzis et al. [17] demonstrated uniform strain at both locations. An advantage of a two-probe procedure is that it eliminates issues with skin markers. However, the probe location used by Arampatzis et al. [17] still involved the use of a marker to cast a shadow onto the field of view as a reference point on the ultrasound image [12, 13]. Skin displacement throughout the activation procedure may potentially influence the calculation of tendon strain by producing an inaccurate measure of tendon resting length [15, 16]. Similar to the present study, Neugebauer and Dawkins [18] positioned two fixed ultrasound probes over the calcaneus and MTJ.

### Variations in activation procedures

Passive elongation or active contraction methods are used to obtain a measure of mechanical deformation (strain). The elongation of the Achilles tendon relative to its resting length is influenced by the chosen activation procedure. In contrast to the present study where Achilles tendon displacement was imaged during passive ankle dorsiflexion, Arampatzis et al. [17] assessed Achilles tendon excursion during a maximal voluntary plantarflexion. Neugebauer and Dawkins [18] obtained tendon excursion measures following a 6-min walk, with imaging occurring during a maximal isometric plantarflexion effort (flexed knee position). Mechanical deformation is influenced by the load applied; when a higher percentage of maximum voluntary contraction is applied, a higher level of strain is produced [34]. Tendon excursion cannot be standardised between passive elongation and maximum voluntary contraction. Therefore, the activation procedure influences the elongation of the Achilles tendon relative to its resting length, ultimately manipulating the calculation of strain.

### Variation in ankle joint range of motion parameters

Ankle joint rotation is an important factor that has been reported to influence elongation of the Achilles tendon because of passive joint movement [13, 33]. Consequently, the effect of Achilles tendon strain through different ranges of motion may explain varying estimations of strain between studies. Arampatzis et al. [17] passively rotated the ankle between 80° and 135° (total range of motion 45°) to obtain elongation of the tendon during passive movement. In comparison, the present study used a range between 80° and 110° (total range of motion 30°). However, the strain measure obtained by Arampatzis et al. [17] cannot be directly compared with our study, because although passive elongation was used to measure the elongation of tendon, the ultrasound images were acquired during a maximum voluntary contraction. During this time, the ankle joint changed from 110° (resting length) to 90°. Consequently, the elongation (and therefore strain) of the Achilles tendon might have been underestimated by Arampatzis et al. [17]. This may explain the difference in the strain value between their study and the present study, where resting length was measured with the ankle positioned at 90°. Neugebauer and Dawkins [18] positioned participants in knee flexion, which is a further possible explanation for the lower strain values in their study than reported in the present study. We positioned participants with their knee in full extension. In knee flexion, the force produced by gastrocnemius is reduced, which may result in underestimation of the strain measurement [2].

### Limitations

This study needs to be considered in light of some limitations. First, participants’ activity levels the day of and day before the procedure were not controlled or standardised between measurement sessions. Second, the ultrasound probe positioned over the calcaneal insertion of the Achilles tendon was manually held. The structure of the posterior calcaneus meant it was not possible to manufacture a custom-made brace to stabilise the probe as was the case for the probe located at the MTJ. Therefore, there might have been some probe movement across the surface of the skin at the calcaneal insertion measurement point. However, the sonographer carefully observed the transducer during data collection to mitigate probe movement. Third, we chose probe locations that would capture a greater length of the tendon; however, the two probe locations (MTJ and insertion) did not examine the entire length of the tendon. Inferences were made about tendon length and strain changes for the whole length from the MTJ and insertion, which are representative of the entire length but not necessarily absolute measures for its entire length.

Finally, we used repeated measures of Achilles tendon strain to quantify reliability in the present study. Given the poor between-session result, there was a possibility that repeated passive elongation might have influenced the viscoelastic properties of the Achilles tendon, therefore directly influencing excursion, elongation and strain measurements.

## Conclusion

We investigated a two-probe procedure for measuring Achilles tendon strain. Data demonstrated excellent within-session intra- and inter-rater reliability; however, the between-session intra-rater reliability was poor. The excellent within-session reliability suggests that once the participant and ultrasound probes are positioned, the two-probe ultrasound imaging procedure is a reliable method to assess Achilles tendon strain. In addition, the methods and software used to calculate strain from pre-captured images are reproducible. Measurement of Achilles tendon strain using a two-probe procedure offers an additional tool for objectively evaluating the mechanical characteristics of the Achilles tendon. However, further reliability trials are warranted to determine which variables contributed to poor between-session reliability and improve the accuracy of this tool in the research environment.

## Data Availability

Request for further details of the data set and queries relating to data sharing arrangements may be submitted to Prue Molyneux (prue.susan.molyneux@aut.ac.nz). Participants were not asked for informed consent for data sharing, although the present data are anonymised and the risk of identification is negligible.

## Abbreviations

CI: Confidence interval
ICC: Intraclass correlation coefficients
MDC: Minimal detectable change
MTJ: Musculotendinous junction
ROI: Region of interest
SD: Standard deviation
SEM: Standard error of measurement
